# Does reporting behaviour bias the measurement of social inequalities in self-rated health in Indonesia? An anchoring vignette analysis

**DOI:** 10.1007/s11136-015-1152-y

**Published:** 2015-10-12

**Authors:** Wulung Hanandita, Gindo Tampubolon

**Affiliations:** Cathie Marsh Institute for Social Research, University of Manchester, Humanities Bridgeford Street Building 2F, Oxford Road, Manchester, M13 9PL UK

**Keywords:** Self-rated health, Socio-economic status, Reporting heterogeneity, Anchoring vignette, Indonesia

## Abstract

**Purpose:**

Studies on self-rated health outcomes are fraught with problems when individuals’ reporting behaviour is systematically biased by demographic, socio-economic, or cultural factors. Analysing the data drawn from the Indonesia Family Life Survey 2007, this paper aims to investigate the extent of differential health reporting behaviour by demographic and socio-economic status among Indonesians aged 40 and older ($N = 3735$).

**Methods:**

Interpersonal heterogeneity in reporting style is identified by asking respondents to rate a number of vignettes that describe varying levels of health status in targeted health domains (mobility, pain, cognition, sleep, depression, and breathing) using the same ordinal response scale that is applied to the self-report health question. A compound hierarchical ordered probit model is fitted to obtain health differences by demographic and socio-economic status. The obtained regression coefficients are then compared to the standard ordered probit model.

**Results:**

We find that Indonesians with more education tend to rate a given health status in each domain more negatively than their less-educated counterparts. Allowing for such differential reporting behaviour results in relatively stronger positive education effects.

**Conclusion:**

There is a need to correct for differential reporting behaviour using vignettes when analysing self-rated health measures in older adults in Indonesia. Unless such an adjustment is made, the salutary effect of education will be underestimated.

## Introduction

Both resource constraints and the multidimensionality of health concepts being studied often necessitate the collection of self-rated health (SRH) data. SRH measures, which ask individuals to report their health status either in general or on a specific health domain using an ordinal response scale, require no specialist intervention during data collection, are relatively cheap and quick to obtain, and are feasible to implement in large-scale surveys. In addition to the belief that SRH can capture aspects of health that cannot be tapped by objective measure [35], research has shown that SRH is highly correlated with assessments provided by health professionals [9] and that is also a strong predictor of mortality [15] as well as health care utilisation [30].

**Fig. 1:**
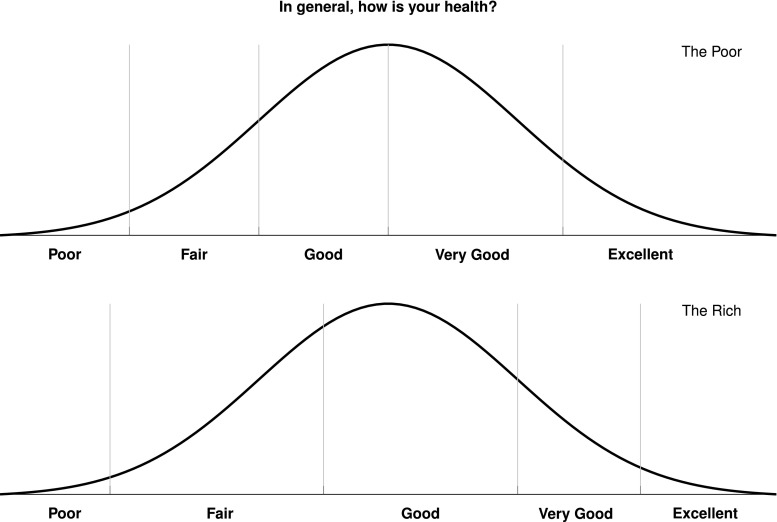
Problem of response-scale heterogeneity

Notwithstanding these benefits, the use of SRH in the study of socio-economic inequalities in health becomes fraught with serious problems when individuals have different expectations, knowledge, or standards of what constitutes a good health. For example, when experiencing an identically severe health problem, poor individuals may paradoxically report better health than their richer counterparts (Fig. 1) simply because the poor have a much higher tolerance to health problems than the rich [28]. This is known in the literature as ‘reporting heterogeneity’ [29], ‘differential item functioning’ [19], ‘response category cut-point shift’ [22], ‘scale of reference bias’ [11], or simply ‘differential reporting’ [20].

To address this problem, the anchoring vignette method has been proposed [18, 19, 32, 36]. By means of this method, researchers can identify the individual-specific reporting style by asking respondents to rate a number of vignettes (hypothetical scenarios) that describe varying levels of health status in a health domain using the same ordinal response scale that is applied to the self-report health. Then, if one is willing to assume that, apart from random error, each vignette is perceived in the same way by all respondents (vignette equivalence assumption) and that they apply exactly the same standard to judge both their own health status and those of the vignettes (response consistency assumption), one can fit a compound hierarchical ordered probit (CHOPIT) model [19] to identify health inequalities that are free from bias due to heterogeneous reporting style.

Using anchoring vignette, it has been shown that among older individuals in eight European countries, there is strong evidence for the existence of differential health reporting by education level. Bago d’Uva et al. [3] found that highly educated older Europeans tend to have higher expectation of health than their less-educated peers and suggested that accounting for differences in the reporting of health is important because ‘measured health inequalities by education are often underestimated, and even go undetected, if no account is taken of these reporting differences’ [3, p. 1375]. However, when the authors analysed data from three most populous developing countries (China, India, and Indonesia), they found that in Indonesia and India, ‘there are either no differences in reporting by education or the better educated are more likely to report very good health’ [2, p. 362]. This finding defies conventional expectation; the authors then speculated that perhaps the Chinese sample, which has a higher level of education than the Indonesian and Indian, were more able to comprehend the vignette exercise.

Motivated by these mixed findings, this paper aims to investigate whether there is evidence for differential reporting behaviour by demographic and socio-economic status (SES) among Indonesians. We analyse the data from the fourth wave of the Indonesia Family Life Survey (IFLS 2007), which is among the very few population studies conducted in developing countries that employed a vignette rating module. The present study departs from the existing application of anchoring vignette method in Indonesia [2] in its use of a newer data set and of fewer and simpler vignettes, as well as in its analysis of a more homogeneous age group.

## Methods

### Study population

The data are drawn from the IFLS 2007, which is a multi-purpose household longitudinal study that collects information from more than 30,000 individuals from 12,000 households living in 260 districts in Indonesia and is representative of about 83 % of the entire population [25]. The IFLS 2007 is the only IFLS wave that has vignette module. Because the module was administered to only a fraction of study participants, the sample of this study is, by design, limited to 3735 adults aged 40 and older. These individuals were asked to report their self-assessment of health, but only one-third of them (1245 individuals) were subjected to the vignette rating questionnaire. Further details regarding sampling and ethical procedure are available in the IFLS’s documentation [25].

### Measures

Survey respondents were asked to evaluate their own health in six health domains (mobility, pain, cognition, sleep, depression, and breathing) using the question ‘Overall in the last 30 days, how much of a problem did you have with $\ldots$?’. Responses were recorded using a five-category ordinal scale: (1) none, (2) mild, (3) moderate, (4) severe, and (5) extreme. In addition to this self-assessment, randomly selected respondents were also asked to evaluate the health status of hypothetical persons described in the vignettes. For each domain, three vignettes of varying severity were presented; respondents were then asked to think about these persons’ experiences as if they were their own and to rate the health status of the persons portrayed in the hypothetical scenarios in the same way they had rated their own health earlier. Vignettes were presented in the order of mild–moderate–severe health problem, and responses were recorded using the same response scale applied to the SRH. For ease of understanding, we reverse-coded the response scale so that a score of 5 represents very good health and a score of 1 represents very poor health.

The SES variables are education (entered as a dummy variable representing those who completed the 9-year compulsory education) and the logarithm of per capita household asset value. We opted to use these SES indicators rather than the usual indicators of income or expenditure because many respondents were already at the retirement age (56 or older). In this case, education is particularly relevant because it is probably the best measure of SES for older adults [12]. In later life, education serves as a good proxy for permanent income and is less endogenous than income as it is usually fixed early in life [12]. Per capita household asset value was measured from the total value of land, property, vehicles, poultry, livestock, fish ponds, hard stem plants, household appliances, household furniture and utensils, savings, deposit, stocks, receivables, and jewellery owned by the household members. Like education, assets are also considered as less endogenous than income due to their accumulative nature [21].

We also include respondents’ age groups (40–49, 50–59, 60–69, 70+), gender, marital status (married and unmarried), family size (dummy variable for those living with more than four household members), and urban or rural residential location.

### Data analysis

For each health domain, we first fit an ordered probit (OPROBIT) model [10] to estimate the effect of demographic and SES variables on health. Then, we refit the same specification with a CHOPIT model [19] that generalises the OPROBIT by allowing cut points or thresholds to be different across individuals.

The CHOPIT model is comprised of two components: the self-assessment and the vignette rating component. In the self-assessment equation, we write the unobserved perceived level of health as:

1 $$\begin{aligned} y_{i}^{*}\sim N(\mu _{i},1) \end{aligned}$$

2 $$\begin{aligned} \mu _{i}= X_{i}\beta \end{aligned}$$

with subscript *i* denotes individuals responding to SRH questionnaire. Individuals’ actual health level $\mu _{i}$ varies as a linear function of observed covariates $X_{i}$ with parameter vector $\beta$. Respondents then turn their perceived level of health $y_{i}^{*}$ into reported ordinal category $y_{i}$ via the following observation mechanism:

3 $$y_{i}=k\quad {\mathrm {if}}\; \tau _{i}^{k-1}<y_{i}^{*}<\tau _{i}^{k},\quad k=1,\ldots ,K$$

where

4 $$\begin{aligned} -\infty =\tau _{i}^{0}<\tau _{i}^{1}<\tau _{i}^{2}<\cdots <\tau _{i}^{K}=\infty \end{aligned}$$

To allow for individual-specific response category cut-point shift, thresholds $\tau _{i}$ are modelled as a linear function of observed covariates $X_{i}$ with parameter vector $\gamma$ and are identified in the model using information obtained from the vignette rating exercise.

5 $$\tau _{i}^{1}= X_{i}\gamma ^{1}$$

6 $$\tau _{i}^{k}= \tau _{i}^{k-1}+X_{i}\gamma ^{k},\quad {\mathrm {for}}\quad k=2,\ldots ,K$$

In the vignette rating equation, we write the perceived level of health of the person described in vignette *j* evaluated by survey respondent *i* as:

7 $$z_{ij}^{*}\sim N(\theta _{j},\sigma ^{2})$$

The actual health level of the person described in the vignette ($\theta _{j}$) is assumed to be identical for every respondent, hence formalising the ‘vignette equivalence’ assumption. As in the self-assessment part of the model, respondents then turn the perceived level of health $z_{ij}^{*}$ into the same *K* ordinal category via similar mechanism:

8 $$\begin{aligned} z_{ij}= & {} k\quad {\mathrm {if}}\;\tau _{ij}^{k-1}<z_{ij}^{*}<\tau _{ij}^{k},\quad k=1,\ldots ,K \end{aligned}$$

Thresholds in the vignette rating equation are determined by the same $\gamma$ parameter as in the self-assessment part, but note that the sample used in each model component need not be identical. The appearance of the same $\gamma$ parameter vector in both self-assessment and vignette rating components thus formalises the ‘response consistency’ assumption.

For identification and model comparability purposes, the standard ordered probit normalisation restriction (intercept is fixed at zero; variance is set to one) [37] is imposed upon both OPROBIT and CHOPIT models. Then, formal tests of reporting homogeneity ($H_{0}{:}\;{\mathrm {all}}\;\gamma =0$) and parallel cut-point shift ($H_{0}{:}\;\gamma ^{1}=\gamma ^{2}=\cdots =\gamma ^{K-1}$) [16] are performed after acquiring the estimate of the CHOPIT model, accompanied by graphical illustrations when necessary. To facilitate interpretation, we also compute the partial effect of relevant variables on the probability of reporting very good health [16].

Only complete observations are used in the modelling exercise, yielding a sample size of 3069 individuals in the SRH equations (82 % of the original sample) and 939–1130 individuals in the vignette rating equations (75–90 % of the original sample).

## Results

**Fig. 2:**
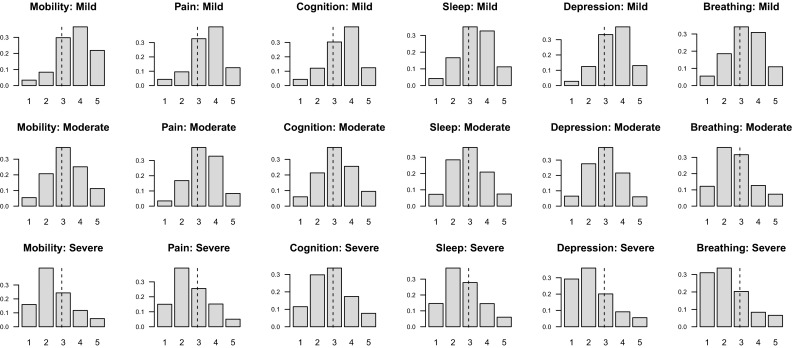
Distribution of vignette ratings (*1* extreme, *2* severe, *3* moderate, *4* mild, *5* none)

We begin with a description of the sample. The mean age is 53.95 (SD = 10.81, median = 52, IQR = 16); half of the sample (52.8 %) are female and 20 % are unmarried. The majority of the sample (77.4 %) live with at least five household members; about half (49.18 %) live in urban area and only one-third (37.92 %) completed the 9-year compulsory education. Per capita household asset value is log-normally distributed with a mean equal to USD 1660 (SD = 3800, median = 721, IQR = 1368). The well-behaved histograms in Fig. 2 show that respondents seem to understand the vignette rating exercise very well: the ratings of moderate health problems are symmetrically distributed, while those of mild and severe health problems are left- and right-skewed, respectively. Overall, there is no marked difference between the characteristics of the SRH sample and those of the vignette sample.

**Fig. 3:**
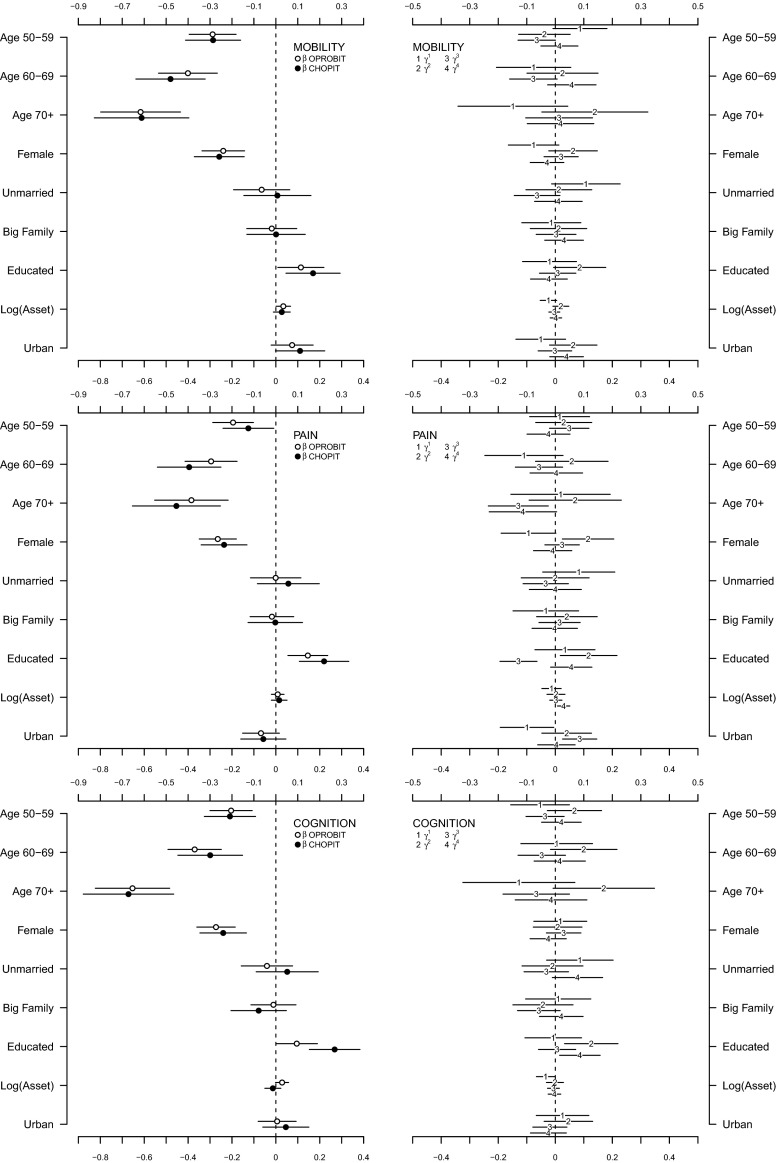
Estimation results for mobility, pain, and cognition domains [main coefficients ($\beta$) in *left panel*, threshold coefficients ($\gamma$) in *right panel*, intercepts in threshold equation not shown]

**Fig. 4:**
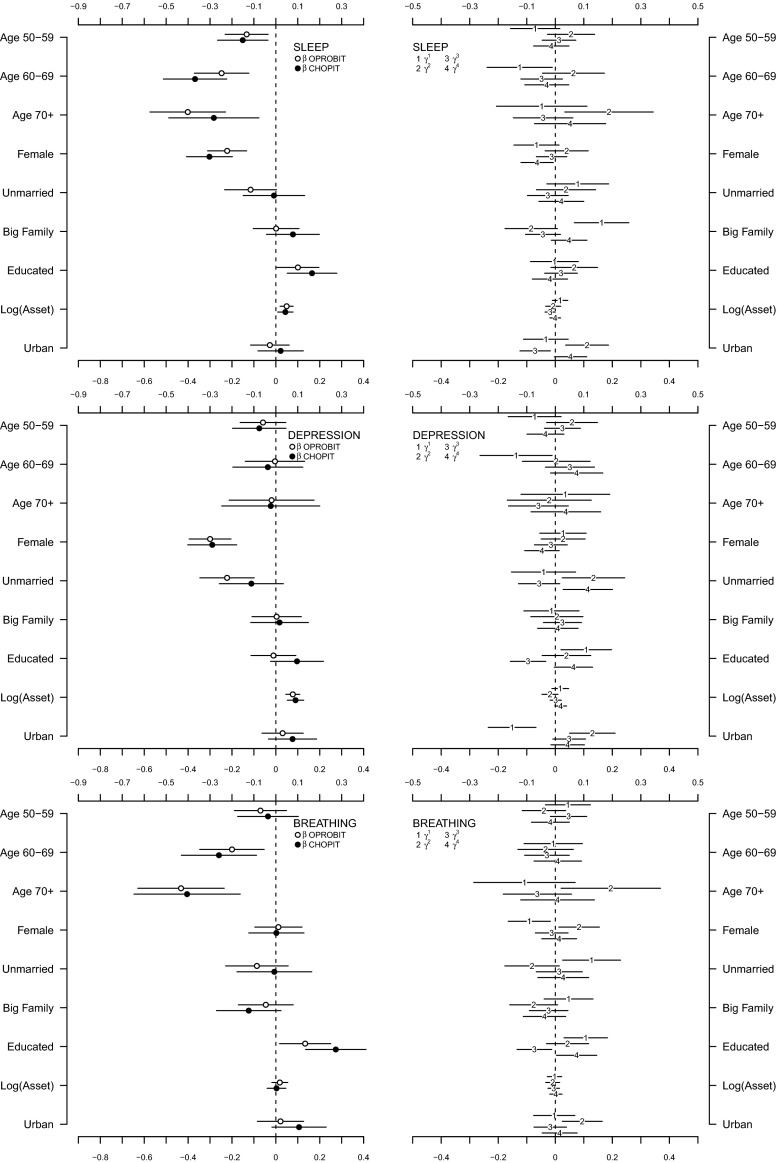
Estimation results for sleep, depression, and breathing domains [main coefficients ($\beta$) in *left panel*, threshold coefficients ($\gamma$) in *right panel*, intercepts in threshold equation not shown]

The regression coefficients obtained from the OPROBIT model are represented by hollow circles plotted in the left panel of both Figs. 3 and 4. Assuming that respondents apply identical thresholds, the results suggest a general trend that (1) health deteriorates with age in a possibly nonlinear fashion (except in the depression domain), (2) women report worse health than men (except in the breathing domain), and (3) the better educated are healthier than those with minimal education attainment (except in the depression domain). Being unmarried is associated with lower health status in the sleep and depression domains, but there is no evidence for such association in other domains. The models show that there seems to be no statistically discernible effect of family size and urban–rural residential location on health in all six domains. Wealth, however, seems to have a positive impact on health in the mobility, cognition, sleep, and depression domains if only to a very small degree. This can be understood as monetary welfare is no longer a good indicator of SES in later life.

**Fig. 5:**
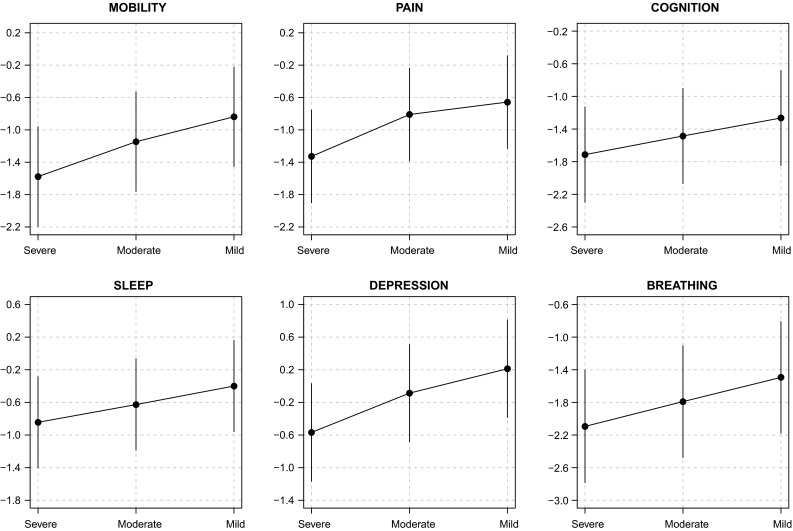
Estimated location of vignette rating $(\theta _{j})$

What happen when we relax the reporting homogeneity assumption by fitting a CHOPIT model? Regression coefficients predicting the latent health index in each domain ($\beta$) are shown using solid circles in the left panels of Figs. 3 and 4, while those predicting the individual-specific thresholds ($\gamma$) are shown using numbers in the right panels of the figures. An omnibus test of reporting homogeneity in each domain (Table 1) rejects the joint null hypothesis that all coefficients in the threshold equation are equal to zero at conventional significance levels, indicating that respondents do not necessarily apply identical cut points when transferring their latent health indices onto the ordinal categories. In other words, there seems to be disagreement as to what constitutes good health among the respondents; some may have higher or lower standards than others. The statistically significant results of a global test of parallel cut-point shift in each domain (except in mobility and cognition; see Table 1) further indicate that respondents’ reporting behaviour depends on the covariates in a complex way. The relationship between the thresholds and the covariates is not necessarily characterised by a simple linear function. Respondents, however, seem to agree on the levels of health described in the vignettes. As shown in Fig. 5, the estimated vignette locations in the latent health space are in concordance with the intended ordering. This confirms the earlier exploratory analysis presented in Fig. 2.

**Table 1:** Test of reporting homogeneity and parallel cut-point shift

Test	Mobility	Pain	Cognition	Sleep	Depression	Breathing
Reporting homogeneity	50.70*	93.86^‡^	82.28^‡^	99.03^‡^	105.46^‡^	98.81^‡^
Parallel cut-point shift	32.16	66.99^‡^	33.40	53.06^‡^	67.98^‡^	46.12^†^

Reported are $\chi ^2$ statistic with 36 degrees of freedom (reporting homogeneity) and 27 degrees of freedom (parallel cut-point shift)

* $$p<0.10; {}^{\dagger}\,p<0.05; {}^{\ddagger}\,p<0.01$$

Allowing for interpersonal differences in reporting style does alter the point estimate of each $\beta$ coefficient (Figs. 3, 4), but with the exception of that of education, the correction is practically negligible. In fact, when we test for reporting homogeneity by each covariate, only education variable is consistently statistically significant in all six health domains (Table 2). After adjusting for reporting heterogeneity, the 95 % confidence intervals of age, gender, family size, wealth, and urban/rural residential location still overlap largely with those of the OPROBIT model, and their interpretation remains. For marital status, the adjustment brings significant change in the sleep and depression domains where the health-protective effect of being married diminishes after correcting for the lower expectation of health among married individuals.

**Table 2:** Test of reporting homogeneity by each covariate

Variable	Mobility	Pain	Cognition	Sleep	Depression	Breathing
Age 50–59						
Age 60–69	${\circledcirc }$	${\circ }$		${\circledcirc }$	${\circledcirc }$	
Age 70+		${\circledcirc }$		${\circledcirc }$		
Female		${\circ }$		${\circledcirc }$		
Unmarried	${\circ }$		${\circ }$	${\circledcirc }$	$\triangle$	${\circ }$
Big family				$\triangle$		
Educated	${\circ }$	$\triangle$	$\triangle$	${\circ }$	$\triangle$	$\triangle$
Log(asset)			$\triangle$			
Urban		${\circ }$		$\triangle$	$\triangle$	$\triangle$

$$^\circ \,p<0.10;\; ^\circledcirc \,p<0.05; \;^\triangle \,p<0.01$$

**Table 3:** Partial effects of education on the probability of reporting very good health

Domain	OPROBIT	CHOPIT
Mobility	0.03 ± 0.01^†^	0.04 ± 0.02^‡^
Pain	0.06 ± 0.02^‡^	0.08 ± 0.02^‡^
Cognition	0.03 ± 0.02^†^	0.09 ± 0.02^‡^
Sleep	0.04 ± 0.02^†^	0.06 ± 0.02^‡^
Depression	−0.00 ± 0.02	0.03 ± 0.02*
Breathing	0.03 ± 0.01^†^	0.06 ± 0.01^‡^

* $$p<0.10;\,{}^{\dagger}\,p<0.05;\,{}^{\ddagger}\,p<0.01$$

**Fig. 6:**
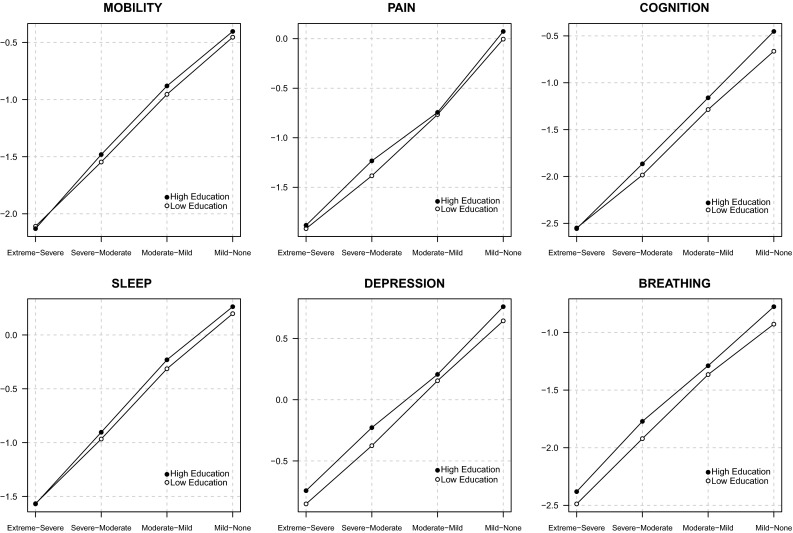
Effect of education on vignette ratings’ cut points

**Fig. 7:**
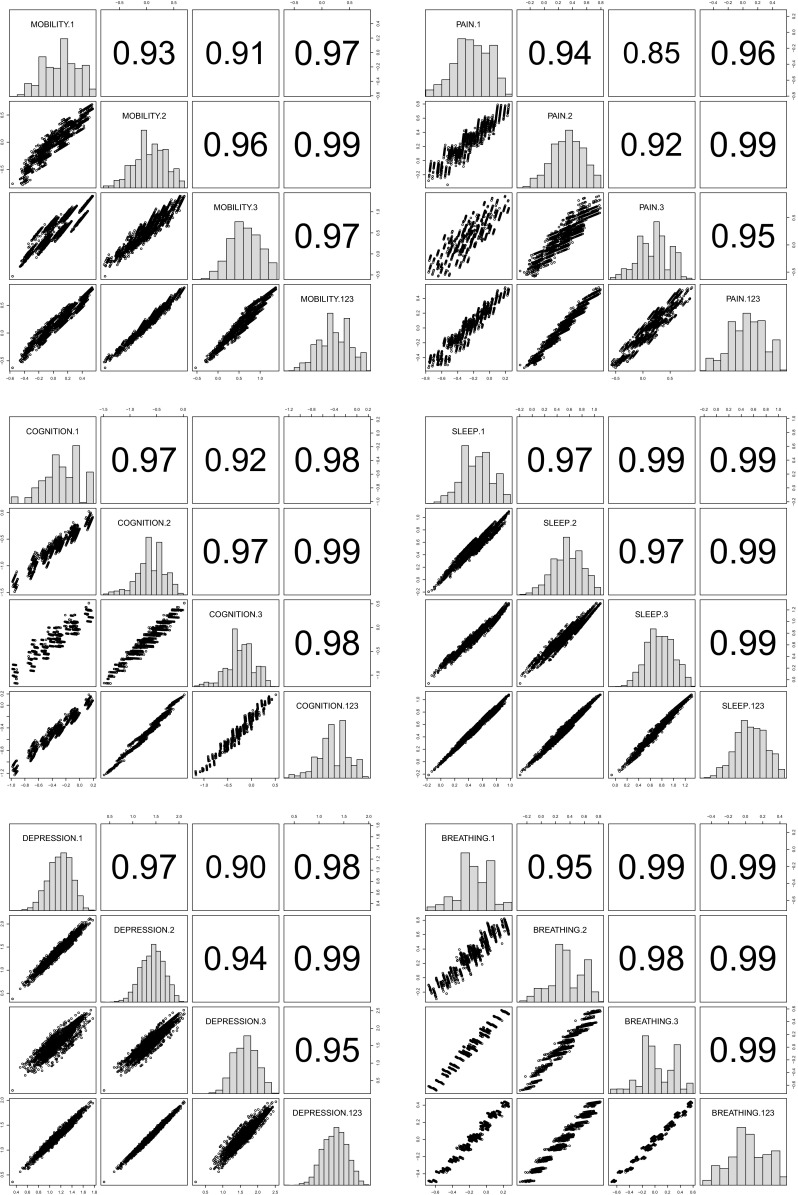
Correlations among pairs of predicted health index in each domain

A significant correction is observed with regard to education. The positive education effect in some threshold equations across health domains (shown in the right panels of Figs. 3, 4) suggests that Indonesians with high levels of educational attainment tend to rate a given health status more negatively than their less-educated counterparts. This is consistent with the educated being better informed; they have higher health standards. Thus, adjusting for this difference magnifies the positive effect of education on health status in all domains (Table 3). Most notably, the adjustment raises the estimated difference in the probability of reporting very good health between the well- and less-educated Indonesians in cognition and breathing domains by two- to threefold. The CHOPIT coefficients also tend to be more precisely estimated. Figure 6 shows how education level alters respondents’ thresholds, which are used to transfer the latent health index onto the ordinal categories. The plots suggest that reporting behaviour depends on education in a rather complex way, reiterating the results of the test of parallel cut-point shift (Tables 1, 4). Finally, following the method of Voňková and Hullegie [34], we test whether or not the adjustment to reporting heterogeneity is sensitive to the choice of vignettes used in the model by refitting the CHOPIT model with a single vignette at a time, predicting the latent health index and then calculating the Pearson's correlation coefficient between pairs of predicted values in each domain. As shown in Fig. 7, the strong correlations suggest that the adjustment is insensitive to the choice of hypothetical scenarios.

## Discussion

Applying anchoring vignette methodology to a sample of older Indonesians, this study investigates the extent of differential reporting behaviour by demographic and socio-economic status in six health domains. We find that allowing for interpersonal heterogeneity in response style consistently magnifies the positive effect of education on health in all domains. One plausible interpretation of this finding is that educated Indonesians, who are likely to be well informed and aware of their well-being, have higher standards or expectations with regard to health than their less-educated counterparts. This indicates that health disparity by education might actually be wider than it is usually reported. Unless an adjustment is made for this systematic differential, the salutary effect of education will be underestimated. This finding is in line with an earlier observation in Europe [3], but it contradicts a previous study showing the overestimation of education effect among the general population in Indonesia [2]. Such a divergence might result from our (1) use of fewer and simpler vignettes, (2) analysis of a more homogeneous age group, and/or (3) use of a newer data set. We also find significant modification in the effect of marital status in the sleep and depression domains. The detrimental effect in these domains of being unmarried diminishes after correcting for the higher expectations of health prevalent among unmarried individuals. Otherwise, we find little difference when calibrating the effects of other demographic variables. Overall, these findings suggest that policy-maker cannot only rely on people’s perception of health when attempting to measure the reality. Studies on self-reported health outcomes particularly in developing countries should consider administering vignettes and using them to arrive at unbiased report on health inequality.

The generalisability of this study is limited by the restricted age group being analysed as well as by the small sample size. Future studies may collect more extensive vignette data so that statistical inferences can be extended to general population and so that stratified analysis by age, gender, or urban/rural residential location can be performed. We also note that the validity of the anchoring vignette method hinges critically on the maintenance of both vignette equivalence and response consistency assumptions. A number of studies have investigated the plausibility of these assumptions; some have found positive supports [19, 26, 31], while others report possible violations [5, 6, 8, 13]. In this study, there is always the possibility that these assumptions are violated. Vignette equivalence, for example, might not hold if some individuals perceive one of the vignettes more as being in a serious health condition because he or she has experienced or taken care of a family member who went through similar illness. Also, unmeasured respondents’ past experience of adverse events, surgery, or major illness, could have an effect on their perception of the vignettes as well as on their response to SRH questionnaire. While we have not provided a direct test for these assumptions, we are at least reassured that our analysis is insensitive to the choice of vignettes used in the model. Furthermore, by asking survey respondents to rate the vignettes as if they assess their own health condition, the IFLS study has at least tried to reinforce the response consistency assumption during data collection stage.

Anchoring vignette is a promising method that offers a direct way of handling interpersonal incomparability in self-report measure. Although methodologists have extended the original anchoring vignette method [19] to accommodate more complex situations [4, 6, 17, 23, 24, 31, 36], adequate attention should also be given to the fundamental matters of question wording [1, 13] and ordering [7, 14]. We believe that, given its cost-effectiveness and feasibility in large-scale surveys, SRH and anchoring vignette have the potential to play a greater role in public health research in now-decentralised Indonesia, where more than 500 local administrations must struggle with a scarcity of competent health workers [27, 33] as well as with the high cost of collecting objective health measures.

## Appendix

### Self-report health question [25]

1. Overall in the last 30 days, how much of a problem did [name of person/you] have with moving around?
2. Overall in the last 30 days, how much of bodily aches or pains did you have?
3. Overall in the last 30 days, how much difficulty did you have with remembering things?
4. In the last 30 days, how much difficulty did you have with sleeping, such as falling asleep, waking up frequently during the night or waking up too early in the morning?
5. Overall in the last 30 days, how much of a problem did you have with feeling sad, low, or depressed?
6. In the last 30 days, how much of a problem did you have because of shortness of breath?

### Vignette description [25]

#### Mobility

1. Pak Taryono/Bu Taryini is able to walk distances of up to 200 metres without any problems but feels tired after walking 1 km. He has no problems with day-to-day activities, such as carrying food from the market.
2. Pak Tumino/Bu Tumini does not exercise. He cannot climb stairs or do other physical activities because he is obese. He is able to carry the groceries and do some light household work.
3. Pak Sidik/Bu Endah has a lot of swelling in his legs due to his health condition. He has to make an effort to walk around his home as his legs feel heavy.

#### Pain

1. Pak Budiarto/Bu Budiarti has a headache once a month that is relieved after taking a pill. During the headache, she can carry on with her day-to-day affairs.
2. Pak Sumarno/Bu Sumarni has pain that radiates down her right arm and wrist during her day at work. This is slightly relieved in the evenings when she is no longer working on her computer.
3. Pak Mulyono/Bu Mulyanti has pain in his knees, elbows, wrists, and fingers, and the pain is present almost all the time. Although medication helps, he feels uncomfortable when moving around, holding, and lifting things.

#### Cognition

1. Pak Taryono/Bu Taryini can concentrate while watching TV, reading a magazine, or playing a game of cards or chess. Once a week, he forgets where his keys or glasses are, but finds them within 5 min.
2. Pak Suwarso/Bu Suwarsih is keen to learn new recipes but finds that she often makes mistakes and has to reread several times before she is able to do them properly.
3. Pak Mugiono/Bu Mugianti cannot concentrate for more than 15 min and has difficulty paying attention to what is being said to him. Whenever he starts a task, he never manages to finish it and often forgets what he was doing. He is able to learn the names of people he meets.

#### Sleep

1. Pak Partono/Bu Partini falls asleep easily at night, but two nights a week she wakes up in the middle of the night and cannot go back to sleep for the rest of the night.
2. Pak Darma/Bu Darmi wakes up almost once every hour during the night. When he wakes up in the night, it takes around 15 min for him to go back to sleep. In the morning, he does not feel well-rested.
3. Pak Parto/Bu Parti takes about 2 h every night to fall asleep. He wakes up once or twice a night feeling panicked and takes more than 1 h to fall asleep again.

#### Depression

1. Pak Arman/Bu Lina enjoys her work and social activities and is generally satisfied with her life. She gets depressed every 3 weeks for a day or two and loses interest in what she usually enjoys but is able to carry on with her day-to-day activities.
2. Pak Sukarso/Bu Sukarsih feels nervous and anxious. He worries and thinks negatively about the future, but feels better in the company of people or when doing something that really interests him. When he is alone, he tends to feel useless and empty.
3. Pak Rano/Bu Rina feels depressed most of the time. She weeps frequently and feels hopeless about the future. She feels that she has become a burden on others and that she would be better dead.

#### Breathing

1. Pak Sugiarto/Bu Suwarsih has no problems while walking slowly. He gets out of breath easily when climbing uphill for 20 m or a flight of stairs.
2. Pak Ramlan/Bu Badriah suffers from respiratory infections about once every year. He is short of breath 3 or 4 times a week and had to be admitted in hospital twice in the past month with a bad cough that required treatment with antibiotics.
3. Pak Hamid/Bu Karsini has been a heavy smoker for 30 years and wakes up with a cough every morning. He gets short of breath even while resting and does not leave the house anymore. He often needs to be put on oxygen.

**Table 4:** Test of parallel cut-point shift by each covariate

Variable	Mobility	Pain	Cognition	Sleep	Depression	Breathing
Age 50–59						
Age 60–69						
Age 70+		${\circledcirc }$		${\circ }$		
Female		${\circ }$		${\circ }$		
Unmarried					$\triangle$	
Big family				${\circ }$		${\circ }$
Educated		$\triangle$	$\triangle$		${\circledcirc }$	${\circledcirc }$
Log(asset)		${\circ }$				
Urban		${\circledcirc }$		$\triangle$	$\triangle$	${\circ }$

$$^\circ \,p<0.10;\; ^\circledcirc \,p<0.05;\; ^\triangle \,p<0.01$$
